# Assessment of Between-Hospital Variation in Readmission and Mortality After Cancer Surgical Procedures

**DOI:** 10.1001/jamanetworkopen.2018.3038

**Published:** 2018-10-05

**Authors:** Sebastien Haneuse, Francesca Dominici, Sharon-Lise Normand, Deborah Schrag

**Affiliations:** 1 Department of Biostatistics, Harvard T.H. Chan School of Public Health, Boston, Massachusetts; 2 Department of Health Care Policy, Harvard Medical School, Boston, Massachusetts; 3 Division of Population Sciences, Dana Farber Cancer Institute, Boston, Massachusetts

## Abstract

**Importance:**

Although current federal quality improvement programs do not include cancer surgery, the Centers for Medicare & Medicaid Services and other payers are considering extending readmission reduction initiatives to include these and other common high-cost episodes.

**Objectives:**

To quantify between-hospital variation in quality-related outcomes and identify hospital characteristics associated with high and low performance.

**Design, Setting, and Participants:**

This retrospective cohort study obtained data through linkage of the California Cancer Registry to hospital discharge claims databases maintained by the California Office of Statewide Health Planning and Development. All 351 acute care hospitals in California at which 1 or more adults underwent curative intent surgery between January 1, 2007, and December 31, 2011, with analyses finalized July 15, 2018, were included. A total of 138 799 adults undergoing surgery for colorectal, breast, lung, prostate, bladder, thyroid, kidney, endometrial, pancreatic, liver, or esophageal cancer within 6 months of diagnosis, with an American Joint Committee on Cancer stage of I to III at diagnosis, were included.

**Main Outcomes and Measures:**

Measures included adjusted odds ratios and variance components from hierarchical mixed-effects logistic regression analyses of in-hospital mortality, 90-day readmission, and 90-day mortality, as well as hospital-specific risk-adjusted rates and risk-adjusted standardized rate ratios for hospitals with a mean annual surgical volume of 10 or more.

**Results:**

Across 138 799 patients at the 351 included hospitals, 8.9% were aged 18 to 44 years and 45.9% were aged 65 years or older, 57.4% were women, and 18.2% were nonwhite. Among these, 1240 patients (0.9%) died during the index admission. Among 137 559 patients discharged alive, 19 670 (14.3%) were readmitted and 1754 (1.3%) died within 90 days. After adjusting for patient case-mix differences, evidence of statistically significant variation in risk across hospitals was identified, as characterized by the variance of the random effects in the mixed model, for all 3 metrics (*P* < .001). In addition, substantial variation was observed in hospital performance profiles: across 260 hospitals with a mean annual surgical volume of 10 or more, 59 (22.7%) had lower-than-expected rates for all 3 metrics, 105 (40.4%) had higher-than-expected rates for 2 of the 3, and 19 (7.3%) had higher-than-expected rates for all 3 metrics.

**Conclusions and Relevance:**

Accounting for patient case-mix differences, there appears to be substantial between-hospital variation in in-hospital mortality, 90-day readmission, and 90-day mortality after cancer surgical procedures. Recognizing the multifaceted nature of hospital performance through consideration of mortality and readmission simultaneously may help to prioritize strategies for improving surgical outcomes.

## Key Points

**Question:**

Is there between-hospital variation in quality metrics for patients undergoing cancer surgery in California?

**Findings:**

In this cohort study of 138 799 patients from 351 California hospitals, substantial between-hospital variation was found in in-hospital mortality, 90-day readmission, and 90-day mortality after cancer surgical procedures.

**Meaning:**

Recognizing the multifaceted nature of hospital performance through consideration of mortality and readmission simultaneously may help to prioritize strategies for improving surgical outcomes.

## Teaser

This cohort study examines the variation between California hospitals in mortality and readmission after cancer surgical procedures.

## Introduction

Hospitals are under increasing scrutiny and financial pressure to publicly report quality-of-care measures.^1^ In 2012, the Hospital Readmissions Reduction Program, established by the Affordable Care Act, tied Medicare hospital reimbursement rates to excess hospital readmissions for patients undergoing select surgical procedures, including elective total hip and/or knee replacement surgery and coronary artery bypass graft surgery.^2,3^

Although current policies do not include cancer surgery, which is the mainstay treatment for most patients with solid tumor malignancies,^4^ the Centers for Medicaid & Medicare Services (CMS) and other payers are considering extending readmission reduction initiatives to include common, high-cost episodes. Previous studies have demonstrated high rates of readmission after common cancer operations and identified reduction as an opportunity to improve quality of care and efficiency.^5,6,7^ However, prior to developing effective quality improvement initiatives for cancer surgery, it is critical to understand between-hospital variation in quality-related outcomes and identify hospital characteristics that are associated with superior or inferior performance. This information could also inform patient decisions regarding where to undergo surgery.

Prior studies have described associations between hospital attributes, particularly case volume, and mortality outcomes of cancer surgery.^8,9,10,11^ In addition, studies have examined the association between hospital characteristics and readmission after cancer surgical procedures.^12,13^ However, to our knowledge, mortality and readmission have not been considered in tandem, which may be problematic because a hospital with low readmission rates and high postoperative mortality rates requires a different quality improvement approach than one with high readmission rates and low postoperative mortality rates.^14,15^ Motivated by these considerations, we quantify between-hospital variation in in-hospital mortality, postdischarge readmission, and postdischarge mortality for patients undergoing primary surgery for early-stage cancer in California.

## Methods

### Data Sources

We conducted a retrospective cohort study using data from the California Cancer Registry linked to hospital discharge records from all California Department of Public Health–licensed health care facilities, maintained by the California Office of Statewide Health Planning and Development. This study was approved by the Dana Farber Cancer Institute Institutional Review Board and the California Department of Public Health. Informed consent was waived for this retrospective, minimal-risk study of deidentified data. Throughout the study, we followed the Strengthening the Reporting of Observational Studies in Epidemiology (STROBE) reporting guideline.^16^

### Study Population

Adults identified in the California Cancer Registry who underwent cancer surgery between January 1, 2007, and December 31, 2011, for 1 of the 11 most common solid tumors (colorectal, breast, lung, prostate, bladder, thyroid, kidney, endometrial, pancreatic, liver, and esophageal) within 6 months of diagnosis were included. Only patients with an American Joint Committee on Cancer (AJCC) stage (6th edition) of I, II, or III at diagnosis were included.^17^ Patients with stage IV tumors at diagnosis or recurrent metastatic cancer were excluded because symptom palliation is typically the primary intent of surgery, rather than cure.

### Outcomes and Performance Measures

We considered in-hospital mortality among all patients, and 90-day and 30-day events for both postdischarge readmission and mortality. Readmission was defined as any admission to a California acute care hospital, regardless of where the patient underwent surgery. Mortality was defined as all-cause mortality. For the postdischarge outcomes, we chose 90-day event rates in the main analysis because the CMS considers 90 days as the duration for delivery of comprehensive care for cancer operations^18^ and studies have advocated this window.^19,20,21,22^

For all 3 outcomes, we report estimates of hospital-specific risk-adjusted rates, based on models that adjust for patient and tumor covariates (see the Statistical Analysis section), as absolute measures of performance. We also report model-based risk-adjusted standardized rate (RASR) ratios for each outcome.^23^ These quantities provide a relative comparison between observed and expected rates for each hospital via internal standardization respect to the specific patients who underwent surgery at the hospital. Thus, a RASR ratio greater than 1.0 may be interpreted as reflecting higher-than-expected outcome rates for the patients who underwent cancer surgery at that hospital, after adjustment for patient-level covariates.

### Hospital Characteristics

Hospital characteristics abstracted from the California Office of Statewide Health Planning and Development databases included ownership type (nonprofit, for-profit, or public), teaching status based on affiliation with a medical school (yes/no), and safety-net hospital status (yes/no) designated if 20% or more of surgical discharges were paid for by the Medi-Cal program. Mean annual hospital surgical volumes for the specified tumors were calculated on the basis of all years (≤5) during which at least 1 surgery was performed and were categorized as 1 to 10, 11 to 50, 51 to 100, 101 to 200, and more than 200. Finally, status as a critical access hospital (yes/no) was ascertained from the California Hospital Association^24^; status as a National Cancer Institute (NCI)-Designated Cancer Center (yes/no) was also ascertained.^25^

### Patient and Tumor Characteristics

Patient demographics included age at surgery, sex, race (white, black, Asian, American Indian, other), and Hispanic ethnicity (yes/no), the primary insurance type/payer (Medicare, Medicaid, commercial, other indigent, self-pay/other/unknown), and whether the admission was scheduled at least 24 hours in advance. A modified Charlson-Deyo comorbidity score (range, 0-25; with higher scores indicating greater comorbidity burden) was calculated using inpatient claims from the California Office of Statewide Health Planning and Development for the 12 months prior to cancer surgery.^26^ Disposition at discharge was categorized as home without services, home with services, skilled nursing/immediate care, and other (including acute care, residential care facility, and other care). Patient socioeconomic status was characterized using the median household income and percentage of people of all ages in poverty in their 2010 census tract residence. Tumor characteristics included primary cancer type and AJCC stage.

### Statistical Analysis

All statistical analyses were performed using R,^27^ version 3.5.0 (R Foundation), and finalized July 15, 2018. Descriptive statistics were calculated for all characteristics. Following methods currently used by the American College of Surgeons Surgical Quality Improvement Program^28^ and the CMS,^23^ for each outcome we fit 2 sets of hierarchical logistic regression models with normally distributed, hospital-specific random effects.^29^ The first set solely included patient and tumor characteristics. The second set additionally included hospital-specific characteristics.

To formally evaluate between-hospital variation in risk, we used a likelihood ratio test for the variance component of the first set of hierarchical logistic regression models that solely adjust for patient and tumor characteristics.^30^ We used the estimated random-effects SD to quantify between-hospital variation in risk and compute the hospital odds ratio (OR), which compares the odds of the outcome for a patient treated at a hospital 1 SD below average quality (when the random effect is 0) to the corresponding odds for the same patient treated at a hospital 1 SD above average quality. Finally, based on the second set of models, we report adjusted ORs and 95% CIs for hospital-specific characteristics.

Estimates of hospital-specific risk adjusted outcome rates were computed as the mean model-based predicted risk across the patients who underwent surgery at the hospital, conditional on the estimated hospital-specific random effect. Hospital-specific RASR ratios were calculated by dividing the estimated hospital-specific risk-adjusted rate by the overall statewide standard for the patients treated at the hospital. The latter quantity was calculated by averaging the hospital-specific rate over the estimated distribution of the random effects. Consistent with current policy, these metrics were calculated on the basis of the fitted hierarchical models that included cancer and patient characteristics.^23^ Although these measures were computed for all 351 hospitals in the study, we only report those for the 260 hospitals with a mean annual surgical volume of 10 or more.

## Results

### Study Sample

Between 2007 and 2011, 138 799 adults were diagnosed with 1 of the 11 tumors we consider at AJCC stages I to III and underwent curative intent surgery at 1 of 351 hospitals in California within 6 months of diagnosis. After surgery, 137 559 patients (99.1%) were discharged alive and 1240 patients (0.9%) died during the index admission (eFigure 1 in the Supplement). Among the 138 799 patients who underwent curative-intent surgery, most had surgery for colorectal, breast, or prostate cancer (20.1%, 21.9%, and 19.7%, respectively), and had an AJCC stage of I or II (78.3%) (Table 1). Among these patients, 8.9% were aged 18 to 44 years and 45.9% were aged 65 years or older. The median age at surgery was 63 years (interquartile range, 54-72 years), with most patients being white (81.8%) or Asian (10.9%) (18.2% were nonwhite) and 57.4% of patients being women. Approximately half of the patients had commercial insurance (48.9%), with most treated at nonprofit (84.0%), nonteaching (78.0%), and non–safety-net (94.1%) hospitals.

**Table 1.** Characteristics of the Study Patients at the Time of Surgery

Characteristic	All Patients		Discharged Alive		
	Overall No. (%)^a^	In-Hospital Mortality, No. (Rate, %)	Overall No. (%)^a^	90-d Readmission, No. (Rate, %)	90-d Mortality, No. (Rate, %)
Total	138 799	1240 (0.9)	137 559	19 670 (14.3)	1754 (1.3)
Cancer type					
Colorectal	27 914 (20.1)	711 (2.5)	27 203 (19.8)	4869 (17.9)	837 (3.1)
Breast	30 378 (21.9)	22 (0.1)	30 356 (22.1)	3706 (12.2)	129 (0.4)
Lung	8885 (6.4)	190 (2.1)	8695 (6.3)	1640 (18.9)	276 (3.2)
Prostate	27 278 (19.7)	11 (0.0)	27 267 (19.8)	1364 (5.0)	41 (0.2)
Bladder	1915 (1.4)	27 (1.4)	1888 (1.4)	650 (34.4)	81 (4.3)
Thyroid	10 530 (7.6)	0 (0.0)	10 530 (7.7)	2878 (27.3)	8 (0.1)
Kidney	13 045 (9.4)	89 (0.7)	12 956 (9.4)	1634 (12.6)	119 (0.9)
Endometrial	14 685 (10.6)	24 (0.2)	14 661 (10.7)	1794 (12.2)	107 (0.7)
Pancreatic	2404 (1.7)	84 (3.5)	2320 (1.7)	685 (29.5)	89 (3.8)
Liver	940 (0.7)	38 (4.0)	902 (0.7)	235 (26.1)	33 (3.7)
Esophageal	825 (0.6)	44 (5.3)	781 (0.6)	215 (27.5)	34 (4.4)
AJCC stage					
I	55 980 (40.3)	287 (0.5)	55 693 (40.5)	7874 (14.1)	425 (0.8)
II	52 661 (37.9)	517 (1.0)	52 144 (37.9)	6385 (12.2)	608 (1.2)
III	30 158 (21.7)	436 (1.4)	29 722 (21.6)	5411 (18.2)	721 (2.4)
Sex					
Men	59 069 (42.6)	717 (1.2)	58 352 (42.4)	7484 (12.8)	827 (1.4)
Women	79 730 (57.4)	523 (0.7)	79 207 (57.6)	12 186 (15.4)	927 (1.2)
Age, y					
18-44	12 374 (8.9)	10 (0.1)	12 364 (9.0)	2263 (18.3)	12 (0.1)
45-54	23 810 (17.2)	40 (0.2)	23 770 (17.3)	3021 (12.7)	68 (0.3)
55-64	38 877 (28.0)	135 (0.3)	38 742 (28.2)	4460 (11.5)	225 (0.6)
65-74	36 361 (26.2)	300 (0.8)	36 061 (26.2)	4890 (13.6)	373 (1.0)
75-84	20 559 (14.8)	451 (2.2)	20 108 (14.6)	3688 (18.3)	623 (3.1)
≥85	6818 (4.9)	304 (4.5)	6514 (4.7)	1348 (20.7)	453 (7.0)
Charlson-Deyo score^b^					
0	120 524 (86.8)	565 (0.5)	119 959 (87.2)	15 006 (12.5)	904 (0.8)
1	8503 (6.1)	188 (2.2)	8315 (6.0)	1835 (22.1)	274 (3.3)
≥2	9772 (7.0)	487 (5.0)	9285 (6.7)	2829 (30.5)	576 (6.2)
Type of admission^c^					
Not scheduled	17 737 (12.8)	632 (3.6)	17 105 (12.4)	3803 (22.2)	743 (4.3)
Scheduled	121 062 (87.2)	608 (0.5)	120 454 (87.6)	15 867 (13.2)	1011 (0.8)
Length of admission, d					
≤1	40 350 (29.1)	68 (0.2)	40 282 (29.3)	4464 (11.1)	70 (0.2)
2-4	56 487 (40.7)	166 (0.3)	56 321 (40.9)	6240 (11.1)	225 (0.4)
5-7	21 620 (15.6)	147 (0.7)	21 473 (15.6)	3527 (16.4)	326 (1.5)
8-14	14 114 (10.2)	289 (2.0)	13 825 (10.1)	3416 (24.7)	529 (3.8)
>14	6228 (4.5)	570 (9.2)	5658 (4.1)	2023 (35.8)	604 (10.7)
Disposition at discharge					
Home without services	NA	NA	117 085 (85.1)	14 508 (12.4)	605 (0.5)
Home with services	NA	NA	13 056 (9.5)	2771 (21.2)	335 (2.6)
Skilled nursing/intermediate care	NA	NA	6235 (4.5)	2035 (32.6)	683 (11.0)
Other	NA	NA	1183 (0.9)	356 (30.1)	131 (11.1)
Marital status					
Not married	53 502 (38.5)	619 (1.2)	52 883 (38.4)	8406 (15.9)	958 (1.8)
Married	85 297 (61.5)	621 (0.7)	84 676 (61.6)	11 264 (13.3)	796 (0.9)
Race					
White	113 511 (81.8)	1031 (0.9)	112 480 (81.8)	16 131 (14.3)	1462 (1.3)
Black	9085 (6.5)	80 (0.9)	9005 (6.5)	1478 (16.4)	137 (1.5)
Asian	15 110 (10.9)	123 (0.8)	14 987 (10.9)	1975 (13.2)	140 (0.9)
American Indian	620 (0.4)	5 (0.8)	615 (0.4)	55 (8.9)	11 (1.8)
Other	473 (0.3)	1 (0.2)	472 (0.3)	31 (6.6)	4 (0.8)
Hispanic ethnicity					
No	116 154 (83.7)	1073 (0.9)	115 081 (83.7)	16 125 (14.0)	1549 (1.3)
Yes	22 645 (16.3)	167 (0.7)	22 478 (16.3)	3545 (15.8)	205 (0.9)
Payer category					
Medicare	57 882 (41.7)	968 (1.7)	56 914 (41.4)	9255 (16.3)	1337 (2.3)
Medicaid	8399 (6.1)	59 (0.7)	8340 (6.1)	1550 (18.6)	97 (1.2)
Commercial	67 856 (48.9)	181 (0.3)	67 675 (49.2)	8128 (12.0)	288 (0.4)
Other indigent	2742 (2.0)	10 (0.4)	2732 (2.0)	429 (15.7)	19 (0.7)
Self-pay/other/unknown	1920 (1.4)	22 (1.1)	1898 (1.4)	308 (16.2)	13 (0.7)
Median income, $					
<50 000	18 653 (13.4)	232 (1.2)	18 421 (13.4)	2497 (13.6)	269 (1.5)
50 000-59 000	75 788 (54.6)	653 (0.9)	75 135 (54.6)	11 249 (15.0)	1005 (1.3)
60 000-69 000	10 664 (7.7)	75 (0.7)	10 589 (7.7)	1426 (13.5)	126 (1.2)
70 000-79 000	22 843 (16.5)	189 (0.8)	22 654 (16.5)	3139 (13.9)	255 (1.1)
≥80 000	10 851 (7.8)	91 (0.8)	10 760 (7.8)	1359 (12.6)	99 (0.9)
% Below poverty line					
<10	10 827 (7.8)	101 (0.9)	10 726 (7.8)	1349 (12.6)	120 (1.1)
10-14	49 944 (36.0)	383 (0.8)	49 561 (36.0)	6674 (13.5)	577 (1.2)
15-19	66 410 (47.8)	604 (0.9)	65 806 (47.8)	10 101 (15.3)	891 (1.4)
≥20	11 618 (8.4)	152 (1.3)	11 466 (8.3)	1546 (13.5)	166 (1.4)
Mean annual patient volume, No.					
0-9	1535 (1.1)	45 (2.9)	1490 (1.1)	289 (19.4)	52 (3.5)
10-49	14 475 (10.4)	199 (1.4)	14 276 (10.4)	2255 (15.8)	281 (2.0)
50-99	19 984 (14.4)	242 (1.2)	19 742 (14.4)	2980 (15.1)	301 (1.5)
100-199	37 539 (27.0)	362 (1.0)	37 177 (27.0)	5250 (14.1)	485 (1.3)
≥200	65 266 (47.0)	392 (0.6)	64 874 (47.2)	8896 (13.7)	635 (1.0)
Hospital type					
Nonprofit	116 648 (84.0)	940 (0.8)	115 708 (84.1)	16 144 (14.0)	1402 (1.2)
For-profit	11 115 (8.0)	197 (1.8)	10 918 (7.9)	1738 (15.9)	205 (1.9)
Public	11 036 (8.0)	103 (0.9)	10 933 (7.9)	1788 (16.4)	147 (1.3)
Teaching hospital					
No	108 215 (78.0)	1051 (1.0)	107 164 (77.9)	14 913 (13.9)	1471 (1.4)
Yes	30 584 (22.0)	189 (0.6)	30 395 (22.1)	4757 (15.7)	283 (0.9)
Safety-net hospital					
No	130 587 (94.1)	1168 (0.9)	129 419 (94.1)	18 092 (14.0)	1650 (1.3)
Yes	8212 (5.9)	72 (0.9)	8140 (5.9)	1578 (19.4)	104 (1.3)
Critical access hospital					
No	138 015 (99.4)	1221 (0.9)	136 794 (99.4)	19 539 (14.3)	1736 (1.3)
Yes	784 (0.6)	19 (2.4)	765 (0.6)	131 (17.1)	18 (2.4)
NCI-designated cancer center					
No	122 721 (88.4)	1165 (0.9)	121 556 (88.4)	17 596 (14.5)	1638 (1.3)
Yes	16 078 (11.6)	75 (0.5)	16 003 (11.6)	2074 (13.0)	116 (0.7)
Year					
2007	24 048 (17.3)	231 (1.0)	23 817 (17.3)	3495 (14.7)	290 (1.2)
2008	29 447 (21.2)	303 (1.0)	29 144 (21.2)	4246 (14.6)	391 (1.3)
2009	29 428 (21.2)	254 (0.9)	29 174 (21.2)	4344 (14.9)	369 (1.3)
2010	28 755 (20.7)	240 (0.8)	28 515 (20.7)	4114 (14.4)	382 (1.3)
2011	27 121 (19.5)	212 (0.8)	26 909 (19.6)	3471 (12.9)	322 (1.2)

Abbreviations: AJCC, American Joint Committee on Cancer; NA, not applicable; NCI, National Cancer Institute.

^a^ Column percentage.

^b^ Range, 0 to 25, with higher scores indicating greater comorbidity burden.

^c^ Arranged with the hospital at least 24 hours prior to the admission.

Of the 137 559 patients who were discharged alive, 19 670 (14.3%) had a readmission within 90 days of discharge (Table 1), and 1754 (1.3%) died within 90 days. At 30 days, these rates were 7.9% and 0.6%, respectively (eTable 1 in the Supplement).

Most hospitals were nonprofit (61.8% [217 of 351]), and only a minority had teaching hospital (7.7% [27 of 351]), safety-net hospital (9.1% [32 of 351]), critical access hospital (6.6% [23 of 351]), and NCI-designated cancer center (2.3% [8 of 351]) status (Table 2). Over the 5-year study period, most hospitals had a mean of 50 or fewer patients per year (57.8% [2013 of 351]), while 39 (11.1%) had a mean of 200 or more patients per year. None of the 92 hospitals with a mean annual surgical volume less than 10 had teaching, safety-net, or NCI-designated cancer center status.

**Table 2.** Characteristics of 351 Hospitals Represented by at Least 1 Patient in the Study Sample and 260 Hospitals With at Least 10 Patients per Year, on Average

Characteristic	No. (%)	
	All Hospitals	Hospitals With ≥10 Patients per Year, on Average
Total	351	260
Hospital type		
Nonprofit	217 (61.8)	179 (68.8)
For-profit	80 (22.8)	46 (17.7)
Public	54 (15.4)	35 (13.5)
Mean annual patient volume, No.		
0-9	91 (25.9)	NA
10-49	112 (31.9)	112 (43.1)
50-99	55 (15.7)	55 (21.2)
100-199	54 (15.4)	54 (20.8)
≥200	39 (11.1)	39 (15.0)
Teaching hospital		
No	324 (92.3)	233 (89.6)
Yes	27 (7.7)	27 (10.4)
Safety-net hospital		
No	319 (90.9)	228 (87.7)
Yes	32 (9.1)	32 (12.3)
Critical access hospital		
No	328 (93.4)	255 (98.1)
Yes	23 (6.6)	5 (1.9)
NCI-designated cancer center		
No	343 (97.7)	252 (96.9)
Yes	8 (2.3)	8 (3.1)

Abbreviations: NA, not applicable; NCI, National Cancer Institute.

### Hierarchical Regression Modeling

Detailed results from the hierarchical logistic regression models are provided in eTables 2, 3, and 4 in the Supplement. After adjusting for patient characteristics, we found evidence of an association between ownership type and in-hospital mortality (*P* < .001) (Table 3). For-profit status of the hospital was associated with 38% higher odds of in-hospital mortality compared with nonprofit status (OR, 1.38; 95% CI, 1.14-1.67); public status of the hospital was associated with 21% higher odds of in-hospital mortality compared with nonprofit status (OR, 1.21; 95% CI, 0.95-1.53). Although we found no evidence of an association between mean annual surgical volume and either 90-day readmission or 90-day mortality, higher mean annual surgical volume was associated with decreased in-hospital mortality (Table 3). Teaching and safety-net status were both associated with increased 90-day readmission (OR, 1.13; 95% CI, 1.03-1.25, and OR, 1.14; 95% CI, 1.01-1.28, respectively), but not with 90-day mortality. The estimated OR associations between status as an NCI-designated cancer center and in-hospital mortality, 90-day readmission and 90-day mortality were 0.81 (95% CI, 0.60-1.09), 0.92 (95% CI, 0.80-1.07) and 0.76 (95% CI, 0.57-1.01), respectively, although none of these reached statistical significance.

**Table 3.** Estimated aORs and 95% CIs for Hospital-Level Characteristics From Hierarchical Logistic Regression Analyses of In-Hospital Mortality, 90-Day Readmission, and 90-Day Mortality^a^

Characteristic	In-Hospital Mortality		90-d Readmission		90-d Mortality	
	aOR (95% CI)	*P* Value^b^	aOR (95% CI)	*P* Value^b^	aOR (95% CI)	*P* Value^b^
Hospital type						
Nonprofit	1 [Reference]	<.001	1 [Reference]	.41	1 [Reference]	.22
For-profit	1.38 (1.14-1.67)		1.05 (0.96-1.15)		0.92 (0.76-1.12)	
Public	1.21 (0.95-1.53)		1.05 (0.95-1.17)		1.18 (0.94-1.47)	
Mean annual patient volume, No.						
0-9	1.30 (0.91-1.87)	.04	1.10 (0.94-1.28)	.58	1.23 (0.87-1.73)	.18
10-49	1 [Reference]		1 [Reference]		1 [Reference]	
50-99	1.05 (0.85-1.29)		1.02 (0.93-1.11)		0.89 (0.73-1.08)	
100-199	1.04 (0.85-1.26)		1.01 (0.93-1.10)		0.87 (0.72-1.05)	
≥200	0.88 (0.71-1.10)		1.06 (0.97-1.17)		0.94 (0.77-1.15)	
Teaching hospital						
No	1 [Reference]	.30	1 [Reference]	.01	1 [Reference]	.11
Yes	0.90 (0.73-1.11)		1.13 (1.03-1.25)		0.87 (0.71-1.07)	
Safety-net hospital						
No	1 [Reference]	.36	1 [Reference]	.03	1 [Reference]	>.99
Yes	0.82 (0.62-1.09)		1.14 (1.01-1.28)		0.97 (0.74-1.26)	
Critical access hospital						
No	1 [Reference]	.45	1 [Reference]	.16	1 [Reference]	.75
Yes	1.23 (0.72-2.09)		1.19 (0.93-1.51)		1.04 (0.59-1.82)	
NCI-designated cancer center						
No	1 [Reference]	.07	1 [Reference]	.29	1 [Reference]	.05
Yes	0.81 (0.60-1.09)		0.92 (0.80-1.07)		0.76 (0.57-1.01)	

Abbreviations: aOR, adjusted odds ratio; NCI, National Cancer Institute.

^a^ Adjusted for cancer type, American Joint Committee on Cancer stage, sex, age, Charlson-Deyo score, marital status, race/ethnicity, whether the admission was scheduled at least 24 hours in advance, disposition at discharge, payer category, and census-based median income and percentage below the poverty line.

^b^ Based on an omnibus Wald test.

After adjusting for patient case-mix, we found evidence of statistically significant variation in risk across hospitals for all 3 outcomes (*P* < .001 based on a likelihood ratio test for the variance component). Comparing a patient who underwent surgery at a hospital 1 SD below average quality (ie, when the hospital-specific random effect is 0) with a patient who underwent surgery at a hospital 1 SD above average quality, after adjusting for differences in patient characteristics between the hospitals, the estimated adjusted hospital OR for in-hospital mortality was 1.62 (95% CI, 1.33-1.98) (eTable 5 in the Supplement). Thus, estimated risk differences for 90-day readmission comparing 2 hospitals that are 2 SDs apart are substantially higher than those observed between teaching and nonteaching hospitals (OR, 1.13) as well as that between safety-net and non–safety-net hospitals (OR, 1.14) (Table 3). The corresponding hospital ORs for 90-day readmission and 90-day mortality were 1.45 (95% CI, 1.37-1.53) and 1.68 (95% CI, 1.44-1.95), respectively (eTable 5 in the Supplement).

### Performance Measures

Across the 260 hospitals with a mean annual surgical volume of 10 or more, model-based in-hospital mortality risk-adjusted rates varied from 0.0% to 4.1% (Figure, A). Furthermore, 90-day readmission and 90-day mortality risk-adjusted rates varied from 7.7% to 23.7% and from 0.1% to 4.2%, respectively. For 42 hospitals (16.1%), the 95% CI for the 90-day readmission risk-adjusted rate was entirely below the statewide overall mean; for 58 hospitals (22.3%), the 95% CI was above the statewide overall rate. Similar results were observed for in-hospital and 90-day mortality.

**Figure.**
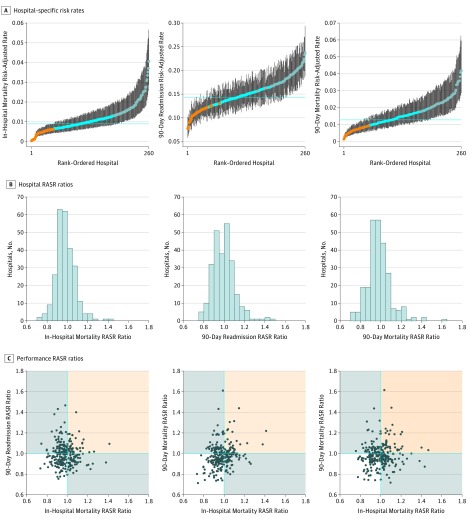
Distributions of Performance Metrics Among 260 Hospitals With at Least 10 Cancer Surgery Patients per Year, on Average, for In-Hospital Mortality, 90-Day Postdischarge Readmission and 90-Day Postdischarge Mortality A, Model-based hospital-specific risk rates and 95% CIs; orange and blue dots indicate that the 95% CI is entirely below and above, respectively, the statewide rate (dotted lines). B, Number of hospitals in discrete categories of model-based risk-adjusted standardized rate (RASR) ratios. C, Bivariate scatterplots for all three 2-way combinations with each point representing a hospital; hospitals in the nonshaded area had optimal performance with RASR ratios less than 1.0, indicating lower-than-expected rates, for both outcomes; hospitals in the orange-shaded region had RASR ratios greater than 1.0, indicating higher-than-expected rates, for both outcomes; hospitals in the gray-shaded region had lower-than-expected rates for 1 metric and higher-than-expected rates for the other.

The distributions of RASR ratios for varied from 0.73 to 1.41 for in-hospital mortality, 0.76 to 1.46 for 90-day readmission, and 0.71 to 1.62 for 90-day mortality (Figure, B). Two-way and 3-way classification of hospitals by RASR ratios indicate a generally positive correlation, but also that there is substantial variation in the hospital-specific profiles (Figure, C and Table 4). Moreover, 7.3% of the hospitals with a mean annual patient volume of more than 10 (19 of 260) had poor performance as indicated by a RASR ratio greater than 1.0 (ie, a higher-than-expected rate) for all 3 outcomes, while 22.7% (59 of 260) had good performance for all 3 outcomes as indicated by a RASR ratio less than1.0 (ie, a lower-than-expected rate) (Table 4). The remaining hospitals had mixed performance, with 40.4% (105 of 260) having good performance for 2 outcomes and 29.6% (77 of 260) having good performance for only 1 outcome.

**Table 4.** Distributions of Performance Profiles, Based on In-Hospital Mortality, 90-Day Readmission, and 90-Day Mortality, Across 260 Hospitals With at Least 10 Cancer Surgery Patients per Year, on Average, Overall, and Stratified by Select Hospital Characteristics^a^

Characteristic	LTE In-Hospital Mortality, No. (%)				HTE In-Hospital Mortality, No. (%)			
	LTE 90-d Mortality		HTE 90-d Mortality		LTE 90-d Mortality		HTE 90-d Mortality	
	LTE 90-d Readmission	HTE 90-d Readmission	LTE 90-d Readmission	HTE 90-d Readmission	LTE 90-d Readmission	HTE 90-d Readmission	LTE 90-d Readmission	HTE 90-d Readmission
Overall (n=260)	59 (22.7)	51 (19.6)	27 (10.4)	29 (11.2)	27 (10.4)	25 (9.6)	23 (8.8)	19 (7.3)
Hospital type								
Nonprofit (n=179)	48 (26.8)	32 (17.9)	18 (10.1)	21 (11.7)	16 (8.9)	15 (8.4)	19 (10.6)	10 (5.6)
For-profit (n=46)	7 (15.2)	11 (23.9)	5 (10.9)	3 (6.5)	6 (13.0)	7 (15.2)	3 (6.5)	4 (8.7)
Public (n=35)	4 (11.4)	8 (2.9)	4 (11.4)	5 (14.3)	5 (14.3)	3 (8.6)	1 (2.9)	5 (14.3)
Mean annual patient volume, No.								
10-49 (n=112)	23 (20.5)	27 (24.1)	12 (10.7)	16 (14.3)	11 (9.8)	6 (5.4)	12 (10.7)	5 (4.5)
50-99 (n=55)	13 (23.6)	10 (18.2)	5 (9.1)	4 (7.3)	8 (14.5)	6 (10.9)	6 (10.9)	3 (5.5)
100-199 (n=54)	13 (24.1)	7 (13.0)	4 (7.4)	3 (5.6)	7 (13.0)	7 (13.0)	4 (7.4)	9 (16.7)
≥200 (n=39)	10 (25.6)	7 (17.9)	6 (15.4)	6 (15.4)	1 (2.6)	6 (15.4)	1 (2.6)	2 (5.1)
Teaching hospital								
No (n=233)	53 (22.7)	44 (18.9)	25 (10.7)	25 (10.7)	26 (11.2)	19 (8.2)	23 (9.9)	18 (7.7)
Yes (n=27)	6 (22.2)	7 (25.9)	2 (7.4)	4 (14.8)	1 (3.7)	6 (22.2)	0	1 (3.7)
Safety-net hospital								
No (n=228)	55 (24.1)	43 (18.9)	23 (10.1)	24 (10.5)	24 (10.5)	20 (8.8)	21 (9.2)	18 (7.9)
Yes (n=32)	4 (2.5)	8 (25.0)	4 (12.5)	5 (15.6)	3 (9.4)	5 (15.6)	2 (6.2)	1 (3.1)

Abbreviations: HTE, higher-than-expected; LTE, lower-than-expected.

^a^ Profiles are based on whether the risk-adjusted standardized rate ratio indicates LTE (risk-adjusted standardized rate ratio <1.0, indicating good performance) or HTE (risk-adjusted standardized rate ratio >1.0, indicating poor performance).

Hospital performance profiles varied across hospital characteristics (Table 4). Among 179 nonprofit hospitals with a mean annual patient volume more than 10, 26.8% (48) had good performance for all 3 outcomes; only 15.2% (7 of 46) and 11.4% (4 of 35) of for-profit and public hospitals, respectively, fell in this optimal category. Similarly, hospitals in this optimal category made up an increasingly large proportion of hospitals as mean annual volume increased (from 20.5% [23 of 112] of hospitals with a volume of 10-49 to 25.6% [10 of 39] of hospitals with a volume ≥200). While teaching and nonteaching hospitals had similar frequencies of suboptimal performers (ie, those with at ≥2 outcomes for which performance was poor), 40.7% (11 of 27) and 36.5% (85 of 233), respectively, they differed substantially in that 66.6% (18 of 27) of teaching hospitals had poor performance with respect to 90-day readmission, while only 45.5% (106 of 233) of nonteaching hospitals had poor performance for 90-day readmission. In contrast, 25.9% (7 of 27) of teaching hospitals and 39.0% (91 of 233) of nonteaching hospitals had poor performance with respect to 90-day mortality. Performance profiles stratified by status as a critical access hospital or as an NCI-designated cancer center are not presented owing to the small number of such hospitals (Table 2).

For postdischarge outcomes, although event rates at 30 days are approximately half those at 90 days, the variation in hospital-specific rates and RASR ratios across hospitals were no different from the 90-day findings (eTable 1 and eFigure 2 in the Supplement).

## Discussion

Although not without controversy,^31^ readmission and mortality are entrenched as hospital quality metrics.^5,12,15,32,33,34,35,36,37^ Although current federal policies focus on a relatively narrow set of conditions, whether readmission and mortality rates are relevant more broadly is the subject of debate.^38,39,40^ In this study, we found substantial and statistically significant variation in these outcomes, as well as in-hospital mortality, across acute care hospitals in California that perform cancer surgery. Furthermore, we found evidence of substantial variation in performance profiles across hospitals, with more than three-quarters of hospitals with higher-than-expected rates for at least 1 of in-hospital mortality, 90-day readmission, and 90-day mortality. Although the absolute rates were lower, findings regarding variation in 30-day readmission and mortality rates were similar.

A crucial feature of our analysis was to consider the 11 cancers simultaneously and, in particular, not to stratify by cancer type. We made this decision for 3 reasons. First, we do not believe that the development and implementation of separate quality improvement initiatives across cancer types represents a viable policy goal. Practically, separate cancer-specific initiatives would likely represent too great a burden on hospitals as well as on decision makers as they seek to reward or penalize high or low quality of care. Furthermore, sample size considerations for a strategy focused on separate initiatives would systematically exclude less-common cancers and low-volume hospitals from potentially benefitting. Second, variation in patient outcomes across cancer types, as well as tumor characteristics, is acknowledged and accounted for in our analyses through their inclusion as adjustment variables in the models. In addition, that there is variation across hospitals in the case-mix of cancer types is the key motivation for reporting the RASR ratio that essentially compares a hospital with itself. Finally, after adjusting for differences in patient case-mix, postoperative quality of care should arguably be agnostic to the procedure, especially after discharge.

Collectively, our results suggest that in-hospital mortality and postdischarge 90-day readmission and mortality are relevant and important targets for the development of tailored interventions and incentive policies regarding quality of care after curative intent surgery. Moreover, although some research has been done on developing and evaluating interventions for reducing readmissions,^41,42^ our results suggest that such interventions and policies should acknowledge the multidimensional nature of quality and target hospitals based on individual performance profiles. For example, policies that encourage careful examination of postdischarge monitoring to anticipate complications and to shift care to the outpatient setting may be appropriate for hospitals with high readmission rates but low mortality rates. In contrast, policies that focus on early recognition of potentially life-threatening postoperative complications, such as sepsis, pulmonary embolism, and dehydration, may be more appropriate for hospitals with high postdischarge mortality. For these hospitals, incentivizing focus on decreasing readmission rates could have the unintended effect of further increasing mortality. From the perspective of individual institutions, simultaneous assessment of RASR ratios across the 3 outcomes may help hospitals better understand their performance with respect to their cancer surgery populations and serve as a useful benchmark for quality improvement initiatives.

A study by Dimick et al^38^ showed that profiling hospital surgical performance on the basis of composite metrics including morbidity, length-of-stay, and reoperation rates can assist hospital performance benchmarking. Our approach did not consider a single composite metric because distinguishing between the 3 events may help hospitals prioritize areas for quality improvement and identify tailored strategies to optimize their performance. As CMS and other health insurers move toward consideration of defined global episodes and outcomes-based reimbursement, we suggest that quality assessments simultaneously consider the in-hospital experience of patients, which is under the direct control of the hospital, together with the vulnerable postdischarge period, which will require hospitals to develop risk-tailored strategies for the frequency of home monitoring and support.

Additional research is needed to better understand heterogeneity in performance profiles across hospitals, in particular why some hospitals perform well on 1 or 2 metrics but not on all 3. In line with other work,^33,43^ we found evidence that certain hospital characteristics are associated with both readmission and mortality, most notably hospital volume. Similar to results presented by Krumholz et al^33^ and Hollis et al,^44^ however, the inclusion of such factors into the hierarchical models only minimally helped explain between-hospital variation. This finding suggests that strategies for improving hospital performance should be customized on the basis of key hospital attributes^43,45,46,47^ as well as their individual performance profiles.

### Limitations

Our study has a number of limitations. First, we did not distinguish between planned and unplanned readmissions, nor did we restrict the analysis to readmissions to the same hospital at which patients were initially treated.^48^ This, however, is in line with the approach currently used by the CMS. Second, some hospitals may have closed or merged during the study period. However, of the 260 hospitals with a mean annual surgical volume of 10 or more, 93.4% contributed to all 5 years of the study period and 95.4% contributed in the final year. Thus, closure and merging would likely have had only minimal influence on characterization of variation in performance across hospitals. Third, our study focused on hospitals in California and we cannot guarantee that the findings generalize to other regions. However, our analyses complement recently published work by Whitney et al,^49^ who used the same data source to describe the high burden of hospitalization within a year of cancer diagnosis.

## Conclusions

After accounting for patient case-mix differences, there is substantial between-hospital variation in in-hospital mortality, 90-day readmission, and 90-day mortality rates among patients undergoing cancer surgery. Policies aimed at improving quality of cancer care should target hospitals based on individual performance profiles.

## Notes

Supplement. **eFigure 1.** Flow Diagram of Patients in the Study **eFigure 2.** Distributions of Performance Metrics Among 260 Hospitals With at Least 10 Cancer Surgery Patients per Year, on Average, for In-Hospital Mortality, 30-Day Post-Discharge Readmission and 90-Day Post-Discharge Mortality **eTable 1.** Characteristics of the Study Patients at the Time of Surgery **eTable 2.** Estimated Adjusted Odds Ratio and 95% Confidence Intervals From Hierarchical Mixed Effects Logistic Regression Analyses for In-Hospital Mortality **eTable 3.** Estimated Adjusted Odds Ratio and 95% Confidence Intervals From Hierarchical Mixed Effects Logistic Regression Analyses for 90-Day Readmission and Mortality **eTable 4.** Estimated Adjusted Odds Ratio and 95% Confidence Intervals From Hierarchical Mixed Effects Logistic Regression Analyses for 30-Day Readmission and Mortality **eTable 5.** Estimates of the Random Effects Standard Deviation (SD), Median Odds Ratio and Hospital Odds Ratio Based on Hierarchical Logistic Regression Analyses of In-Hospital Mortality and 30- and 90-Day Post-Discharge Readmission and Mortality
