# Supplementary material for: Assessment of Between-Hospital Variation in Readmission and Mortality After Cancer Surgical Procedures
Source: JAMA Netw Open. 2018 Oct 5;1(6):e183038. doi: 10.1001/jamanetworkopen.2018.3038 (PMC6324436; doi:10.1001/jamanetworkopen.2018.3038)
Supplement: Supplement. — eFigure 1. Flow Diagram of Patients in the Study eFigure 2. Distributions of Performance Metrics Among 260 Hospitals With at Least 10 Cancer Surgery Patients per Year, on Average, for In-Hospital Mortality, 30-Day Post-Discharge Readmission and 90-Day Post-Discharge Mortality eTable 1. Characteristics of the Study Patients at the Time of Surgery eTable 2. Estimated Adjusted Odds Ratio and 95% Confidence Intervals From Hierarchical Mixed Effects Logistic Regression Analyses for In-Hospital Mortality eTable 3. Estimated Adjusted Odds Ratio and 95% Confidence Intervals From Hierarchical Mixed Effects Logistic Regression Analyses for 90-Day Readmission and Mortality eTable 4. Estimated Adjusted Odds Ratio and 95% Confidence Intervals From Hierarchical Mixed Effects Logistic Regression Analyses for 30-Day Readmission and Mortality eTable 5. Estimates of the Random Effects Standard Deviation (SD), Median Odds Ratio and Hospital Odds Ratio Based on Hierarchical Logistic Regression Analyses of In-Hospital Mortality and 30- and 90-Day Post-Discharge Readmission and Mortality [file jamanetwopen-1-e183038-s001.pdf]

Supplementary Online Content

Haneuse S, Dominici F, Normand S-L, Schrag D. Assessment of between-hospital variation in readmission and mortality after cancer surgical procedures. *JAMA Netw Open*. 2018;1(6):e183038. doi:10.1001/jamanetworkopen.2018.3038

eFigure 1. Flow Diagram of Patients in the Study

eFigure 2. Distributions of Performance Metrics Among 260 Hospitals With at Least 10 Cancer Surgery Patients per Year, on Average, for In-Hospital Mortality, 30-Day Post-Discharge Readmission and 90-Day Post-Discharge Mortality

eTable 1. Characteristics of the Study Patients at the Time of Surgery

eTable 2. Estimated Adjusted Odds Ratio and 95% Confidence Intervals From Hierarchical Mixed Effects Logistic Regression Analyses for In-Hospital Mortality

eTable 3. Estimated Adjusted Odds Ratio and 95% Confidence Intervals From Hierarchical Mixed Effects Logistic Regression Analyses for 90-Day Readmission and Mortality

eTable 4. Estimated Adjusted Odds Ratio and 95% Confidence Intervals From Hierarchical Mixed Effects Logistic Regression Analyses for 30-Day Readmission and Mortality

eTable 5. Estimates of the Random Effects Standard Deviation (SD), Median Odds Ratio and Hospital Odds Ratio Based on Hierarchical Logistic Regression Analyses of In-Hospital Mortality and 30- and 90-Day Post-Discharge Readmission and Mortality

This supplementary material has been provided by the authors to give readers additional information about their work.

© 2018 Haneuse S et al. *JAMA Network Open.*

eFigure 1. Flow Diagram of Patients in the Study.

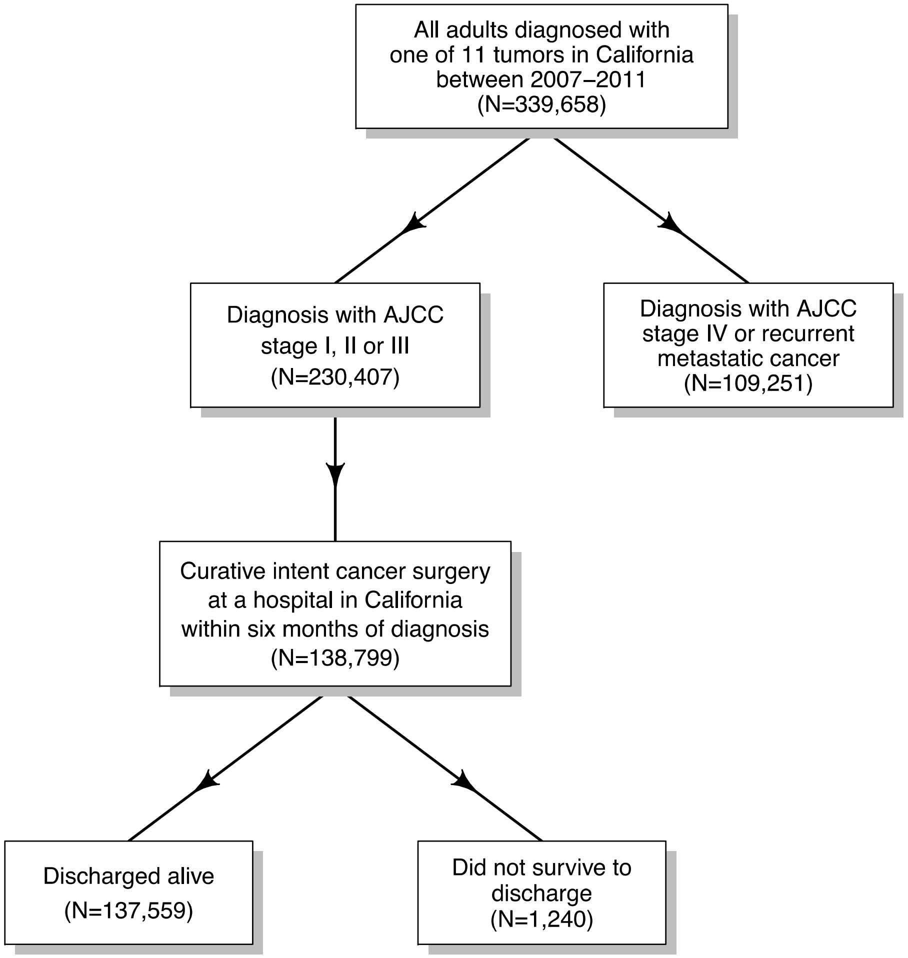

© 2018 Haneuse S et al. *JAMA Network Open.*

eFigure 2. Distributions of Performance Metrics Among 260 Hospitals With at Least 10 Cancer Surgery Patients per Year, on Average, for In-Hospital Mortality, 30-Day Post-Discharge Readmission and 90-Day Post-Discharge Mortality. The top row presents model-based hospital-specific risk rates and 95% confidence intervals (CI); green/red dots indicate that the 95% CI is entirely below/above, and red dots entirely above, the statewide rate. The middle row displays the number of hospitals in discrete categories of model-based risk-adjusted standardized rate (RASR) ratios. The bottom row presents bivariate scatter-plots for all three two-way combinations with each point representing a hospital; hospitals in the white (non-shaded) area had optimal performance with RASR ratios < 1.0, indicating lower than expected rates, for both outcomes; hospitals in the dark shaded region had RASR ratios > 1.0, indicating higher than expected rates, for both outcomes; hospitals in the light shaded region had lower than expected rates for one metric and higher than expected rates for the other.

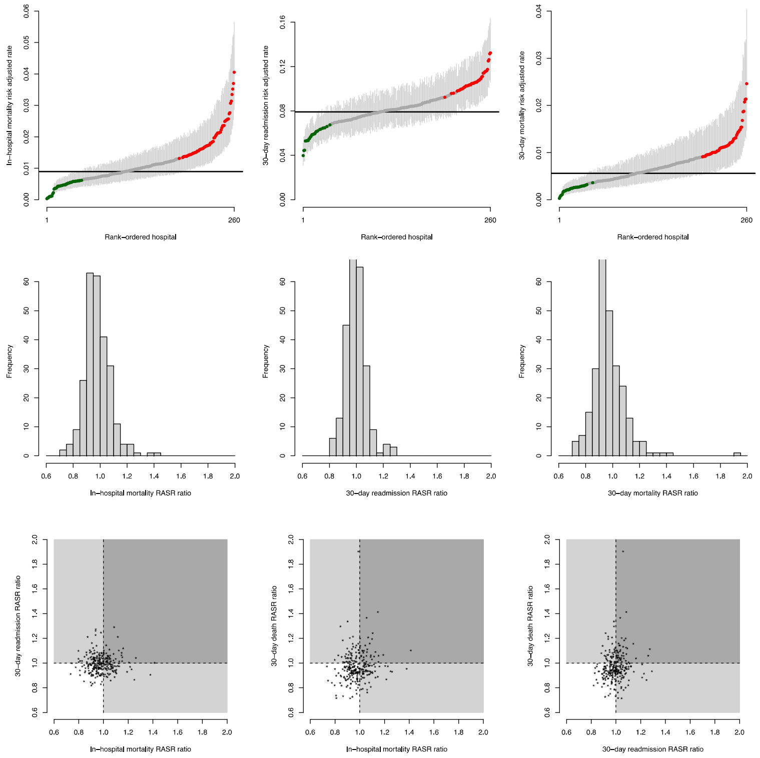

© 2018 Haneuse S et al. *JAMA Network Open.*

eTable 1. Characteristics of the Study Patients at the Time of Surgery

	Discharged alive					
	Overall		30-day readmission		30-day mortality	
	N	%[a]	N	Rate, %	N	Rate, %
**Total**	137,559		10,880	7.9	766	0.6
**Cancer type**						
Colorectal	27,203	19.8	3,031	11.1	401	1.5
Breast	30,356	22.1	1,645	5.4	32	0.1
Lung	8,695	6.3	978	11.2	115	1.3
Prostate	27,267	19.8	981	3.6	26	0.1
Bladder	1,888	1.4	453	24.0	24	1.3
Thyroid	10,530	7.7	990	9.4	2	0.0
Kidney	12,956	9.4	994	7.7	62	0.5
Endometrium	14,661	10.7	1,035	7.1	45	0.3
Pancreas	2,320	1.7	470	20.3	31	1.3
Liver	902	0.7	163	18.1	13	1.4
Esophagus	781	0.6	140	17.9	15	1.9
**AJCC stage**						
Stage I	55,693	40.5	4,258	7.6	203	0.4
Stage II	52,144	37.9	3,728	7.1	261	0.5
Stage III	29,722	21.6	2,894	9.7	302	1.0

© 2018 Haneuse S et al. *JAMA Network Open.*

		Discharged alive							
		**Overall**			**30-day readmission**			**30-day mortality**	
		N	%[a]		N	Rate, %		N	Rate, %
**Sex**									
Male		58,352	42.4		4,721	8.1		391	0.7
Female		79,207	57.6		6,159	7.8		375	0.5
**Age, yrs**									
18-44		12,364	9.0		973	7.9		3	0.0
45-54		23,770	17.3		1,514	6.4		23	0.1
55-64		38,742	28.2		2,567	6.6		97	0.3
65-74		36,061	26.2		2,818	7.8		146	0.4
75-84		20,108	14.6		2,195	10.9		276	1.4
85+		6,514	4.7		813	12.5		221	3.4
**Charlson-Deyo score[b]**									
0		119,959	87.2		8,084	6.7		385	0.3
1		8,315	6.0		1,097	13.2		119	1.4
2+		9,285	6.7		1,699	18.3		262	2.8
**Type of admission[c]**									
Not scheduled		17,105	12.4		2,182	12.8		332	1.9
Scheduled		120,454	87.6		8,698	7.2		434	0.4
**Length of admission, days**									
≤ 1		40,282	29.3		1,978	4.9		20	0.0

© 2018 Haneuse S et al. *JAMA Network Open.*

		Discharged alive						
		Overall			30-day readmission			30-day mortality	
		N	%[a]		N	Rate, %		N	Rate, %
2-4		56,321	40.9		3,286	5.8		97	0.2
5-7		21,473	15.6		2,126	9.9		122	0.6
8-14		13,825	10.1		2,161	15.6		222	1.6
> 14		5,658	4.1		1,329	23.5		305	5.4
**Disposition at discharge**									
Home without services		117,085	85.1		7,681	6.6		224	0.2
Home with services		13,056	9.5		1,687	12.9		144	1.1
Skilled nursing/Intermediate care		6,235	4.5		1,294	20.8		328	5.3
Other		1,183	0.9		218	18.4		70	5.9
**Marital status**									
Not Married		52,883	38.4		4,646	8.8		440	0.8
Married		84,676	61.6		6,234	7.4		326	0.4
**Race**									
White		112,480	81.8		8,914	7.9		631	0.6
Black		9,005	6.5		863	9.6		72	0.8
Asian		14,987	10.9		1,050	7.0		57	0.4
American Indian		615	0.4		35	5.7		4	0.7
Other		472	0.3		18	3.8		2	0.4

© 2018 Haneuse S et al. *JAMA Network Open.*

		Discharged alive							
		**Overall**			**30-day readmission**			**30-day mortality**	
		N	%[a]		N	Rate, %		N	Rate, %
**Hispanic**									
No		115,081	83.7		9,043	7.9		680	0.6
Yes		22,478	16.3		1,837	8.2		86	0.4
**Payer category**									
Medicare		56,914	41.4		5,417	9.5		599	1.1
Medicaid		8,340	6.1		842	10.1		41	0.5
Commercial		67,675	49.2		4,224	6.2		114	0.2
Other Indigent		2,732	2.0		219	8.0		7	0.3
Self Pay/Other/Unknown		1,898	1.4		178	9.4		5	0.3
**Median income ($1,000)**									
< 50		18,421	13.4		1,384	7.5		127	0.7
50-59		75,135	54.6		6,164	8.2		443	0.6
60-69		10,589	7.7		816	7.7		52	0.5
70-79		22,654	16.5		1,722	7.6		100	0.4
80+		10,760	7.8		794	7.4		44	0.4
**Percent below poverty line**									
< 10		10,726	7.8		765	7.1		55	0.5
10-14		49,561	36.0		3,792	7.7		231	0.5
15-19		65,806	47.8		5,497	8.4		407	0.6

© 2018 Haneuse S et al. *JAMA Network Open.*

		Discharged alive						
		Overall			30-day readmission			30-day mortality	
		N	%[a]		N	Rate, %		N	Rate, %
20+		11,466	8.3		826	7.2		73	0.6
**Average annual patient volume**									
0-9		1,490	1.1		169	11.3		26	1.7
10-49		14,276	10.4		1,253	8.8		132	0.9
50-99		19,742	14.4		1,600	8.1		134	0.7
100-199		37,177	27.0		2,884	7.8		201	0.5
200+		64,874	47.2		4,974	7.7		273	0.4
**Hospital type**									
Non-profit		115,708	84.1		8,945	7.7		606	0.5
For-profit		10,918	7.9		964	8.8		92	0.8
Public		10,933	7.9		971	8.9		68	0.6
**Teaching hospital**									
No		107,164	77.9		8,225	7.7		656	0.6
Yes		30,395	22.1		2,655	8.7		110	0.4
**Safety-net hospital**									
No		129,419	94.1		10,051	7.8		721	0.6
Yes		8,140	5.9		829	10.2		45	0.6
**Critical access hospital**									
No		136,794	99.4		10,806	7.9		756	0.6

© 2018 Haneuse S et al. *JAMA Network Open.*

		Discharged alive						
		**Overall**			**30-day readmission**			**30-day mortality**	
		N	%[a]		N	Rate, %		N	Rate, %
Yes		765	0.6		74	9.7		10	1.3
**NCI-designated cancer center**									
No		121,556	88.4		9,607	7.9		726	0.6
Yes		16,003	11.6		1,273	8.0		40	0.2
**Year**									
2007		23,817	17.3		1,889	7.9		112	0.5
2008		29,144	21.2		2,314	7.9		176	0.6
2009		29,174	21.2		2,390	8.2		155	0.5
2010		28,515	20.7		2,264	7.9		175	0.6
2011		26,909	19.6		2,023	7.5		148	0.6

[a] Column percentage

[b] Range 0-25, with higher scores indicating greater co-morbidity burden.

[c] Arranged with the hospital at least 24 hours prior to the admission

© 2018 Haneuse S et al. *JAMA Network Open.*

eTable 2. Estimated Adjusted Odds Ratio (aOR) and 95% Confidence Intervals (CI) From Hierarchical Mixed Effects Logistic Regression Analyses for In-Hospital Mortality. Model 1 includes patient and tumor characteristics. Model 2 additionally includes hospital-level characteristics.

		**In-hospital mortality**			
		**Model 1**		**Model 2**	
		aOR	95% CI	aOR	95% CI
**Cancer type**					
Colorectum		REF		REF	
Breast		0.10	(0.07, 0.16)	0.10	(0.07, 0.16)
Lung		2.01	(1.66, 2.42)	2.06	(1.7, 2.48)
Prostate		0.05	(0.02, 0.08)	0.05	(0.03, 0.09)
Bladder		0.76	(0.51, 1.14)	0.84	(0.56, 1.26)
Thyroid		0.00	-	0.00	-
Kidney		0.72	(0.57, 0.92)	0.75	(0.59, 0.95)
Endometrium		0.31	(0.2, 0.47)	0.32	(0.21, 0.49)
Pancreas		2.57	(1.99, 3.31)	2.80	(2.17, 3.62)
Liver		3.64	(2.51, 5.28)	4.17	(2.87, 6.06)
Esophagus		4.57	(3.25, 6.44)	4.80	(3.41, 6.75)
**AJCC stage**					
Stage I		REF		REF	
Stage II		1.70	(1.45, 2)	1.71	(1.45, 2.01)
Stage III		1.92	(1.63, 2.27)	1.94	(1.65, 2.29)

© 2018 Haneuse S et al. *JAMA Network Open.*

		In-hospital mortality			
		**Model 1**		**Model 2**	
		aOR	95% CI	aOR	95% CI
**Sex**					
Male		REF		REF	
Female		0.61	(0.54, 0.7)	0.62	(0.54, 0.7)
**Age, yrs**					
18-44		REF		REF	
45-54		1.34	(0.67, 2.7)	1.33	(0.66, 2.69)
55-64		2.41	(1.26, 4.6)	2.39	(1.25, 4.57)
65-74		3.76	(1.95, 7.22)	3.66	(1.9, 7.05)
75-84		6.42	(3.33, 12.35)	6.23	(3.23, 12)
85+		10.41	(5.36, 20.19)	10.10	(5.2, 19.62)
**Charlson-Deyo score[a]**					
0		REF		REF	
1		1.40	(1.17, 1.67)	1.40	(1.18, 1.67)
2+		2.65	(2.31, 3.04)	2.67	(2.33, 3.06)
**Type of admission[b]**					
Not scheduled		REF		REF	
Scheduled		0.38	(0.33, 0.44)	0.40	(0.35, 0.46)
**Marital status**					
Not Married		REF		REF	
Married		0.88	(0.77, 0.99)	0.88	(0.78, 1)

© 2018 Haneuse S et al. *JAMA Network Open.*

		In-hospital mortality			
		**Model 1**		**Model 2**	
		aOR	95% CI	aOR	95% CI
**Race**					
White		REF		REF	
Black		1.11	(0.87, 1.41)	1.11	(0.87, 1.42)
Asian		0.95	(0.77, 1.17)	0.94	(0.77, 1.16)
American Indian		1.16	(0.46, 2.89)	1.13	(0.45, 2.82)
Other		0.41	(0.06, 2.99)	0.38	(0.05, 2.79)
**Hispanic**					
No		REF		REF	
Yes		0.96	(0.8, 1.14)	0.96	(0.8, 1.15)
**Payer category**					
Medicare		REF		REF	
Medicaid		0.97	(0.72, 1.31)	0.98	(0.72, 1.32)
Commercial		0.78	(0.63, 0.97)	0.78	(0.63, 0.96)
Other Indigent		0.64	(0.33, 1.23)	0.64	(0.33, 1.25)
Self Pay/Other/Unknown		1.41	(0.89, 2.24)	1.39	(0.87, 2.21)
**Median income ($1,000)**					
< 50		1.07	(0.82, 1.41)	1.03	(0.8, 1.33)
50-59		REF		REF	
60-69		0.83	(0.6, 1.15)	0.84	(0.62, 1.15)
70-79		1.03	(0.79, 1.34)	1.00	(0.78, 1.28)

© 2018 Haneuse S et al. *JAMA Network Open.*

		In-hospital mortality			
		**Model 1**		**Model 2**	
		aOR	95% CI	aOR	95% CI
80+		1.18	(0.85, 1.66)	1.13	(0.83, 1.54)
**Percent below poverty line**					
< 10		1.06	(0.81, 1.38)	1.11	(0.86, 1.43)
10-14		REF		REF	
15-19		1.17	(0.94, 1.47)	1.16	(0.94, 1.43)
20+		1.67	(1.15, 2.44)	1.69	(1.19, 2.4)
**Hospital type**					
Non-profit				REF	
For-profit				1.38	(1.14, 1.67)
Public				1.21	(0.95, 1.53)
**Average annual patient volume**					
0-9				1.30	(0.91, 1.87)
10-49				REF	
50-99				1.05	(0.85, 1.29)
100-199				1.04	(0.85, 1.26)
200+				0.88	(0.71, 1.1)
**Teaching hospital**					
No				REF	
Yes				0.90	(0.73, 1.11)

© 2018 Haneuse S et al. *JAMA Network Open.*

		In-hospital mortality			
		Model 1		Model 2	
		aOR	95% CI	aOR	95% CI
**Safety-net hospital**					
No				REF	
Yes				0.82	(0.62, 1.09)
**Critical access hospital**					
No				REF	
Yes				1.23	(0.72, 2.09)
**NCI-designated cancer center**					
No				REF	
Yes				0.81	(0.6, 1.09)
[a] Range 0-25, with higher scores indicating greater co-morbidity burden.					
[b] Arranged with the hospital at least 24 hours prior to the admission					

© 2018 Haneuse S et al. *JAMA Network Open.*

eTable 3. Estimated Adjusted Odds Ratio and 95% Confidence Intervals From Hierarchical Mixed Effects Logistic Regression Analyses for 90-Day Readmission and Mortality. Model 1 includes patient and tumor characteristics. Model 2 additionally includes hospital-level characteristics.

	**90-day readmission**				**90-day mortality**			
	**Model 1**		**Model 2**		**Model 1**		**Model 2**	
	aOR	95% CI	aOR	95% CI	aOR	95% CI	aOR	95% CI
**Cancer type**								
Colorectum	REF		REF		REF		REF	
Breast	0.97	(0.92, 1.03)	0.97	(0.92, 1.03)	0.54	(0.44, 0.66)	0.54	(0.44, 0.66)
Lung	1.33	(1.24, 1.42)	1.33	(1.24, 1.42)	2.40	(2.03, 2.83)	2.47	(2.09, 2.92)
Prostate	0.33	(0.31, 0.36)	0.33	(0.31, 0.36)	0.21	(0.15, 0.29)	0.21	(0.15, 0.3)
Bladder	2.14	(1.92, 2.39)	2.14	(1.91, 2.38)	1.72	(1.32, 2.23)	1.82	(1.4, 2.37)
Thyroid	2.93	(2.75, 3.13)	2.93	(2.74, 3.13)	0.21	(0.11, 0.43)	0.22	(0.11, 0.44)
Kidney	0.88	(0.83, 0.94)	0.88	(0.83, 0.94)	0.89	(0.72, 1.1)	0.92	(0.75, 1.14)
Endometrium	1.08	(1.01, 1.16)	1.08	(1.01, 1.16)	1.11	(0.88, 1.4)	1.14	(0.9, 1.43)
Pancreas	2.06	(1.86, 2.28)	2.05	(1.85, 2.27)	2.42	(1.88, 3.11)	2.56	(1.99, 3.3)
Liver	1.81	(1.54, 2.13)	1.80	(1.53, 2.12)	3.20	(2.15, 4.74)	3.51	(2.36, 5.22)
Esophagus	1.66	(1.40, 1.96)	1.65	(1.39, 1.95)	2.48	(1.7, 3.63)	2.58	(1.76, 3.77)
**AJCC stage**								
Stage I	REF		REF		REF		REF	
Stage II	1.22	(1.17, 1.28)	1.22	(1.17, 1.28)	1.48	(1.28, 1.7)	1.48	(1.29, 1.71)
Stage III	1.53	(1.47, 1.60)	1.53	(1.47, 1.6)	2.49	(2.17, 2.84)	2.49	(2.18, 2.85)

© 2018 Haneuse S et al. *JAMA Network Open.*

		**90-day readmission**					**90-day mortality**			
		**Model 1**		**Model 2**			**Model 1**		**Model 2**	
		aOR	95% CI	aOR	95% CI		aOR	95% CI	aOR	95% CI
**Sex**										
Male		REF		REF			REF		REF	
Female		0.88	(0.85, 0.92)	0.89	(0.85, 0.92)		0.75	(0.67, 0.85)	0.75	(0.67, 0.84)
**Age, yrs**										
18-44		REF		REF			REF		REF	
45-54		0.85	(0.79, 0.9)	0.85	(0.79, 0.9)		1.99	(1.07, 3.68)	1.99	(1.07, 3.69)
55-64		0.84	(0.79, 0.89)	0.84	(0.79, 0.89)		3.51	(1.95, 6.31)	3.51	(1.95, 6.31)
65-74		0.83	(0.77, 0.9)	0.84	(0.78, 0.9)		4.01	(2.21, 7.27)	4.00	(2.21, 7.25)
75-84		0.85	(0.78, 0.93)	0.86	(0.79, 0.93)		6.92	(3.81, 12.57)	6.90	(3.8, 12.53)
85+		0.81	(0.73, 0.9)	0.81	(0.74, 0.9)		11.21	(6.14, 20.49)	11.14	(6.1, 20.37)
**Charlson-Deyo score**[a]										
0		REF		REF			REF		REF	
1		1.39	(1.31, 1.47)	1.39	(1.31, 1.47)		1.33	(1.15, 1.54)	1.33	(1.14, 1.54)
2+		2.00	(1.89, 2.11)	2.00	(1.9, 2.11)		1.96	(1.73, 2.22)	1.96	(1.73, 2.21)
**Type of admission**[b]										
Not scheduled		REF		REF			REF		REF	
Scheduled		0.85	(0.81, 0.89)	0.85	(0.81, 0.89)		0.59	(0.52, 0.67)	0.59	(0.52, 0.67)
**Disposition at discharge**										
Home without services		REF		REF			REF		REF	
Home with services		1.46	(1.38, 1.53)	1.46	(1.39, 1.54)		1.91	(1.65, 2.21)	1.92	(1.65, 2.22)

© 2018 Haneuse S et al. *JAMA Network Open.*

		90-day readmission					90-day mortality			
		**Model 1**		**Model 2**			**Model 1**		**Model 2**	
		aOR	95% CI	aOR	95% CI		aOR	95% CI	aOR	95% CI
Skilled nursing/Intermediate care		2.21	(2.07, 2.36)	2.22	(2.08, 2.37)		5.34	(4.65, 6.12)	5.33	(4.64, 6.11)
Other		2.04	(1.79, 2.33)	2.04	(1.79, 2.33)		6.98	(5.61, 8.68)	6.92	(5.56, 8.61)
**Marital status**										
Not Married		REF		REF			REF		REF	
Married		1.03	(1, 1.07)	1.03	(1, 1.07)		0.94	(0.84, 1.04)	0.94	(0.84, 1.05)
**Race**										
White		REF		REF			REF		REF	
Black		1.17	(1.1, 1.25)	1.17	(1.1, 1.25)		1.31	(1.08, 1.59)	1.30	(1.06, 1.58)
Asian		0.83	(0.79, 0.88)	0.83	(0.78, 0.88)		0.83	(0.68, 1)	0.82	(0.68, 1)
American Indian		0.60	(0.45, 0.79)	0.60	(0.45, 0.79)		2.17	(1.14, 4.12)	2.14	(1.12, 4.07)
Other		0.46	(0.31, 0.67)	0.46	(0.32, 0.67)		1.25	(0.44, 3.5)	1.24	(0.44, 3.47)
**Hispanic**										
No		REF		REF			REF		REF	
Yes		1.05	(1, 1.1)	1.04	(1, 1.09)		0.87	(0.74, 1.02)	0.86	(0.73, 1.01)
**Payer category**										
Medicare		REF		REF			REF		REF	
Medicaid		1.08	(1, 1.17)	1.05	(0.97, 1.14)		1.32	(1.03, 1.68)	1.32	(1.03, 1.69)
Commercial		0.86	(0.81, 0.91)	0.86	(0.82, 0.91)		0.85	(0.71, 1.02)	0.86	(0.72, 1.02)
Other Indigent		0.87	(0.77, 0.99)	0.84	(0.74, 0.95)		0.88	(0.54, 1.44)	0.89	(0.54, 1.46)

© 2018 Haneuse S et al. *JAMA Network Open.*

		90-day readmission					90-day mortality			
		**Model 1**		**Model 2**			**Model 1**		**Model 2**	
		aOR	95% CI	aOR	95% CI		aOR	95% CI	aOR	95% CI
Self Pay/Other/Unknown		0.94	(0.82, 1.08)	0.92	(0.8, 1.06)		0.73	(0.41, 1.3)	0.73	(0.41, 1.3)
**Median income ($1,000)**										
< 50		0.89	(0.81, 0.98)	0.91	(0.82, 1)		0.95	(0.74, 1.21)	0.94	(0.74, 1.2)
50-59		REF		REF			REF		REF	
60-69		1.07	(0.97, 1.19)	1.08	(0.97, 1.2)		0.89	(0.68, 1.16)	0.88	(0.67, 1.15)
70-79		1.11	(1.01, 1.22)	1.11	(1.01, 1.21)		0.84	(0.66, 1.06)	0.85	(0.68, 1.07)
80+		1.04	(0.92, 1.17)	1.04	(0.92, 1.16)		0.80	(0.59, 1.09)	0.80	(0.59, 1.09)
**Percent below poverty line**										
< 10		0.92	(0.85, 1)	0.93	(0.86, 1.01)		0.96	(0.75, 1.23)	0.96	(0.76, 1.23)
10-14		REF		REF			REF		REF	
15-19		1.13	(1.04, 1.23)	1.13	(1.04, 1.22)		1.04	(0.86, 1.26)	1.05	(0.87, 1.27)
20+		1.15	(1, 1.31)	1.13	(0.99, 1.29)		1.28	(0.91, 1.8)	1.25	(0.9, 1.76)
**Hospital type**										
Non-profit				REF					REF	
For-profit				1.05	(0.96, 1.15)				0.92	(0.76, 1.12)
Public				1.05	(0.95, 1.17)				1.18	(0.94, 1.47)
**Average annual patient volume**										
0-9				1.10	(0.94, 1.28)				1.23	(0.87, 1.73)

© 2018 Haneuse S et al. *JAMA Network Open.*

		90-day readmission					90-day mortality			
		**Model 1**		**Model 2**			**Model 1**		**Model 2**	
		aOR	95% CI	aOR	95% CI		aOR	95% CI	aOR	95% CI
10-49				REF					REF	
50-99				1.02	(0.93, 1.11)				0.89	(0.73, 1.08)
100-199				1.01	(0.93, 1.1)				0.87	(0.72, 1.05)
200+				1.06	(0.97, 1.17)				0.94	(0.77, 1.15)
**Teaching hospital**										
No				REF					REF	
Yes				1.13	(1.03, 1.25)				0.87	(0.71, 1.07)
**Safety-net hospital**										
No				REF					REF	
Yes				1.14	(1.01, 1.28)				0.97	(0.74, 1.26)
**Critical access hospital**										
No				REF					REF	
Yes				1.19	(0.93, 1.51)				1.04	(0.59, 1.82)
**NCI-designated cancer center**										
No				REF					REF	
Yes				0.92	(0.8, 1.07)				0.76	(0.57, 1.01)

[a] Range 0-25, with higher scores indicating greater co-morbidity burden.

[b] Arranged with the hospital at least 24 hours prior to the admission

© 2018 Haneuse S et al. *JAMA Network Open.*

eTable 4. Estimated Adjusted Odds Ratio and 95% Confidence Intervals From Hierarchical Mixed Effects Logistic Regression Analyses for 30-Day Readmission and Mortality. Model 1 includes patient and tumor characteristics. Model 2 additionally includes hospital-level characteristics.

	**30-readmission**				**30-day mortality**			
	**Model 1**		**Model 2**		**Model 1**		**Model 2**	
	aOR	95% CI	aOR	95% CI	aOR	95% CI	aOR	95% CI
**Cancer type**								
Colorectum	REF		REF		REF		REF	
Breast	0.68	(0.63, 0.73)	0.68	(0.63, 0.73)	0.30	(0.21, 0.45)	0.30	(0.21, 0.45)
Lung	1.16	(1.07, 1.26)	1.16	(1.07, 1.26)	1.85	(1.45, 2.37)	1.93	(1.51, 2.48)
Prostate	0.40	(0.36, 0.44)	0.40	(0.36, 0.43)	0.30	(0.2, 0.47)	0.32	(0.2, 0.5)
Bladder	2.19	(1.94, 2.47)	2.17	(1.92, 2.45)	0.96	(0.62, 1.5)	1.06	(0.68, 1.65)
Thyroid	1.33	(1.21, 1.45)	1.32	(1.21, 1.45)	0.13	(0.03, 0.54)	0.14	(0.03, 0.56)
Kidney	0.83	(0.77, 0.9)	0.83	(0.77, 0.9)	0.97	(0.72, 1.3)	1.01	(0.76, 1.36)
Endometrium	0.96	(0.88, 1.04)	0.95	(0.87, 1.04)	1.01	(0.71, 1.42)	1.04	(0.74, 1.48)
Pancreas	2.08	(1.85, 2.33)	2.05	(1.83, 2.31)	1.64	(1.1, 2.44)	1.78	(1.2, 2.66)
Liver	1.86	(1.54, 2.23)	1.83	(1.52, 2.2)	2.40	(1.32, 4.38)	2.79	(1.52, 5.13)
Esophagus	1.58	(1.3, 1.92)	1.57	(1.29, 1.91)	2.06	(1.18, 3.57)	2.16	(1.24, 3.76)
**AJCC stage**								
Stage I	REF		REF		REF		REF	
Stage II	1.09	(1.03, 1.15)	1.09	(1.03, 1.15)	1.24	(1, 1.52)	1.24	(1.01, 1.53)
Stage III	1.26	(1.19, 1.33)	1.26	(1.19, 1.33)	1.93	(1.58, 2.35)	1.94	(1.59, 2.36)

© 2018 Haneuse S et al. *JAMA Network Open.*

		30-readmission					30-day mortality			
		**Model 1**		**Model 2**			**Model 1**		**Model 2**	
		aOR	95% CI	aOR	95% CI		aOR	95% CI	aOR	95% CI
**Sex**										
Male		REF		REF			REF		REF	
Female		0.84	(0.8, 0.89)	0.84	(0.8, 0.89)		0.65	(0.55, 0.77)	0.64	(0.54, 0.76)
**Age, yrs**										
18-44		REF		REF			REF		REF	
45-54		0.85	(0.78, 0.93)	0.85	(0.78, 0.93)		2.46	(0.74, 8.25)	2.47	(0.74, 8.27)
55-64		0.89	(0.82, 0.97)	0.89	(0.82, 0.97)		5.30	(1.67, 16.84)	5.30	(1.67, 16.82)
65-74		0.85	(0.77, 0.94)	0.86	(0.77, 0.95)		5.17	(1.61, 16.58)	5.12	(1.6, 16.44)
75-84		0.89	(0.8, 1)	0.90	(0.81, 1)		9.74	(3.03, 31.27)	9.63	(3, 30.91)
85+		0.87	(0.76, 0.99)	0.87	(0.77, 0.99)		16.16	(5, 52.23)	15.95	(4.94, 51.54)
**Charlson-Deyo score**[a]										
0		REF		REF			REF		REF	
1		1.35	(1.26, 1.46)	1.35	(1.26, 1.46)		1.27	(1.01, 1.58)	1.27	(1.01, 1.58)
2+		1.81	(1.69, 1.93)	1.81	(1.7, 1.93)		1.79	(1.49, 2.15)	1.79	(1.49, 2.15)
**Type of admission**[b]										
Not scheduled		REF		REF			REF		REF	
Scheduled		0.89	(0.84, 0.95)	0.90	(0.85, 0.95)		0.68	(0.56, 0.81)	0.68	(0.57, 0.82)

© 2018 Haneuse S et al. *JAMA Network Open.*

		30-readmission					30-day mortality			
		**Model 1**		**Model 2**			**Model 1**		**Model 2**	
		aOR	95% CI	aOR	95% CI		aOR	95% CI	aOR	95% CI
**Disposition at discharge**										
Home without services		REF		REF			REF		REF	
Home with services		1.47	(1.38, 1.56)	1.47	(1.38, 1.57)		2.42	(1.93, 3.05)	2.44	(1.94, 3.07)
Skilled nursing/Intermediate care		2.19	(2.03, 2.37)	2.20	(2.03, 2.37)		6.75	(5.47, 8.32)	6.74	(5.47, 8.32)
Other		1.97	(1.69, 2.3)	1.96	(1.68, 2.29)		9.67	(7.16, 13.06)	9.61	(7.11, 12.99)
**Marital status**										
Not Married		REF		REF			REF		REF	
Married		1.02	(0.97, 1.06)	1.02	(0.98, 1.06)		0.83	(0.71, 0.98)	0.84	(0.71, 0.98)
**Race**										
White		REF		REF			REF		REF	
Black		1.22	(1.13, 1.33)	1.22	(1.13, 1.32)		1.59	(1.22, 2.08)	1.57	(1.2, 2.05)
Asian		0.83	(0.78, 0.89)	0.83	(0.77, 0.89)		0.82	(0.61, 1.1)	0.82	(0.61, 1.09)
American Indian		0.74	(0.53, 1.05)	0.74	(0.52, 1.05)		1.71	(0.61, 4.79)	1.66	(0.59, 4.68)
Other		0.54	(0.34, 0.88)	0.54	(0.34, 0.88)		1.45	(0.34, 6.08)	1.42	(0.34, 5.95)

© 2018 Haneuse S et al. *JAMA Network Open.*

		30-readmission					30-day mortality			
		**Model 1**		**Model 2**			**Model 1**		**Model 2**	
		aOR	95% CI	aOR	95% CI		aOR	95% CI	aOR	95% CI
**Hispanic**										
No		REF		REF			REF		REF	
Yes		1.03	(0.97, 1.09)	1.02	(0.96, 1.08)		0.87	(0.68, 1.1)	0.86	(0.67, 1.09)
**Payer category**										
Medicare		REF		REF			REF		REF	
Medicaid		1.17	(1.06, 1.29)	1.13	(1.02, 1.24)		1.35	(0.94, 1.94)	1.35	(0.93, 1.96)
Commercial		0.86	(0.8, 0.93)	0.87	(0.81, 0.93)		0.81	(0.62, 1.06)	0.81	(0.62, 1.06)
Other Indigent		0.88	(0.75, 1.03)	0.84	(0.71, 0.98)		0.72	(0.33, 1.6)	0.73	(0.33, 1.63)
Self Pay/Other/Unknown		1.02	(0.86, 1.2)	0.98	(0.83, 1.17)		0.69	(0.28, 1.7)	0.69	(0.28, 1.71)
**Median income ($1,000)**										
< 50		0.93	(0.83, 1.03)	0.95	(0.85, 1.05)		1.22	(0.88, 1.69)	1.22	(0.88, 1.69)
50-59		REF		REF			REF		REF	
60-69		1.03	(0.92, 1.16)	1.04	(0.93, 1.16)		0.87	(0.59, 1.29)	0.87	(0.59, 1.29)
70-79		1.05	(0.95, 1.16)	1.04	(0.94, 1.14)		0.78	(0.56, 1.1)	0.80	(0.57, 1.12)
80+		1.07	(0.94, 1.21)	1.05	(0.93, 1.19)		0.82	(0.53, 1.28)	0.83	(0.54, 1.28)
**Percent below poverty line**										
< 10		0.89	(0.81, 0.98)	0.91	(0.82, 1)		1.13	(0.8, 1.61)	1.15	(0.81, 1.63)

© 2018 Haneuse S et al. *JAMA Network Open.*

		30-readmission					30-day mortality			
		**Model 1**		**Model 2**			**Model 1**		**Model 2**	
		aOR	95% CI	aOR	95% CI		aOR	95% CI	aOR	95% CI
10-14		REF		REF			REF		REF	
15-19		1.07	(0.98, 1.17)	1.06	(0.98, 1.16)		1.11	(0.85, 1.46)	1.12	(0.86, 1.47)
20+		1.03	(0.89, 1.2)	1.01	(0.87, 1.17)		1.12	(0.7, 1.8)	1.08	(0.68, 1.72)
**Hospital type**										
Non-profit				REF					REF	
For-profit				1.06	(0.97, 1.17)				0.94	(0.71, 1.23)
Public				1.08	(0.97, 1.2)				1.28	(0.94, 1.74)
**Average annual patient volume**										
0-9				1.09	(0.91, 1.32)				1.27	(0.79, 2.02)
10-49				REF					REF	
50-99				0.98	(0.89, 1.07)				0.88	(0.67, 1.15)
100-199				0.98	(0.9, 1.07)				0.82	(0.63, 1.06)
200+				1.05	(0.95, 1.15)				0.99	(0.75, 1.32)
**Teaching hospital**										
No				REF					REF	
Yes				1.09	(1, 1.19)				0.80	(0.6, 1.07)
**Safety-net hospital**										
No				REF					REF	
Yes				1.13	(1, 1.27)				0.99	(0.68, 1.44)

© 2018 Haneuse S et al. *JAMA Network Open.*

		30-readmission					30-day mortality			
		**Model 1**		**Model 2**			**Model 1**		**Model 2**	
		aOR	95% CI	aOR	95% CI		aOR	95% CI	aOR	95% CI
**Critical access hospital**										
No				REF					REF	
Yes				1.21	(0.91, 1.59)				1.13	(0.55, 2.35)
**NCI-designated cancer center**										
No				REF					REF	
Yes				1.01	(0.89, 1.14)				0.64	(0.42, 0.98)

[a] Range 0-25, with higher scores indicating greater co-morbidity burden.

[b] Arranged with the hospital at least 24 hours prior to the admission

© 2018 Haneuse S et al. *JAMA Network Open.*

eTable 5. Estimates of the Random Effects Standard Deviation (SD), Median Odds Ratio and Hospital Odds Ratio[a] Based on Hierarchical Logistic Regression Analyses of In-Hospital Mortality and 30- and 90-Day Post-Discharge Readmission and Mortality

		**Patient and census-based characteristics only**			**Patient, census-based and hospital-specific characteristics**	
		**Estimate**	**95% CI**		**Estimate**	**95% CI**
**In-hospital mortality**						
Random effects SD		0.24	(0.14, 0.34)		0.11	(0.00, 0.29)
Median odds ratio		1.26	(1.15, 1.39)		1.11	(0.94, 1.31)
Hospital odds ratio[a]		1.62	(1.33, 1.98)		1.24	(0.87, 1.77)
**30-day readmission**						
Random effects SD		0.14	(0.11, 0.18)		0.13	(0.09, 0.16)
Median odds ratio		1.15	(1.11, 1.18)		1.13	(1.09, 1.16)
Hospital odds ratio[a]		1.33	(1.24, 1.43)		1.29	(1.21, 1.38)
**30-day mortality**						
Random effects SD		0.30	(0.18, 0.43)		0.25	(0.12, 0.38)
Median odds ratio		1.34	(1.19, 1.50)		1.27	(1.12, 1.44)
Hospital odds ratio[a]		1.84	(1.44, 2.34)		1.66	(1.28, 2.15)
**90-day readmission**						
Random effects SD		0.18	(0.16, 0.21)		0.17	(0.14, 0.20)
Median odds ratio		1.19	(1.16, 1.22)		1.17	(1.15, 1.20)
Hospital odds ratio[a]		1.45	(1.37, 1.53)		1.40	(1.33, 1.48)

© 2018 Haneuse S et al. *JAMA Network Open.*

		Patient and census-based characteristics only			Patient, census-based and hospital-specific characteristics	
		Estimate	95% CI		Estimate	95% CI
**90-day mortality**						
Random effects SD		0.26	(0.18, 0.33)		0.23	(0.15, 0.31)
Median odds ratio		1.28	(1.19, 1.38)		1.25	(1.16, 1.34)
Hospital odds ratio[a]		1.68	(1.44, 1.95)		1.58	(1.36, 1.85)

[a] Odds ratio comparing risk of the outcome between a hospital one standard deviation above the (statewide) average to a hospital one standard deviation below the average

© 2018 Haneuse S et al. *JAMA Network Open.*
